# Message Framing Effects on Individuals' Social Distancing and Helping Behavior During the COVID-19 Pandemic

**DOI:** 10.3389/fpsyg.2021.579164

**Published:** 2021-03-22

**Authors:** Melis Ceylan, Ceren Hayran

**Affiliations:** ^1^Faculty of Business Administration, Bilkent University, Ankara, Turkey; ^2^School of Business, Ozyegin University, Istanbul, Turkey

**Keywords:** COVID-19, message framing, prosocial motives, self-interested motives, helping, experiment, social distancing and stay-at-home orders

## Abstract

This research responds to urgent calls to fill knowledge gaps on COVID-19 (new coronavirus) in communicating social distancing messages to the public in the most convincing ways. The authors explore the effectiveness of framing social distancing messages around prosocial vs. self-interested appeals in driving message compliance and helping behavior. The results show that when a message emphasizes benefits for everyone in society, rather than solely for the individual, citizens find the message more persuasive to engage in social distancing, and also more motivating to help others. The results further demonstrate that the proposed effects are higher for individuals who have a lower locus of control and lower fear of coronavirus as prosocial messages lead them to feel a joint responsibility in protecting from the pandemic. Theoretical and practical implications of the results are discussed.

## Introduction

Identified at the beginning of 2020, the COVID-19 (new coronavirus) outbreak has become a global health crisis. Coronavirus is characterized as highly contagious because of its fast spread rate around the world. Prevention has become specifically important because of a lack of approved treatments and vaccines at the early stage of the pandemic. The World Health Organization (WHO, 2020a) has announced a set of preventive measures, among which are attention to personal hygiene, frequent hand washing, social distancing, and self-isolation. Because coronavirus is transmitted through close contact among people, keeping a distance from others has been the key means to curb the spread of the disease. Authorities have been imposing social distancing rules at varying degrees, from suspending public gatherings to more restrictive lockdown orders to minimize interactions among people. Nevertheless, it has been challenging to persuade individuals to comply with distancing messages (Gunia, 2020; Pinsker, 2020). A collective effort is needed to prevent further community spread of the virus, yet little is known about what kind of public messages is most effective in motivating individuals to follow suit. In this respect, the first goal of this research is to examine how citizens respond to subtle changes in the framing of social distancing messages. Specifically, we explore whether implying an individual's own well-being (by using self-interested appeals) or everyone's well-being in the community (by using prosocial appeals) is more persuasive in encouraging message compliance.

COVID-19 has created detrimental social and economic consequences. Many people have lost their jobs and encountered financial difficulties and mental and physical problems. Economically underprivileged societies have faced increased poverty and inadequate healthcare (UNDP United Nations Development Program, 2020). Parties varying from health professionals (Spector, 2020) to academics (Marston et al., 2020) and non-profit organizations have been emphasizing the importance of community support in the fight against COVID-19. To the best of our knowledge, previous research has not explored the factors that promote citizens' willingness to help others during the pandemic. Thus, the second aim of this research is to understand how different messages can encourage helping behavior. More specifically, we compare the causal effect of using self-interested vs. prosocial appeals in a social distancing message on motivating individuals' tendency to help one another.

There has been an increasing academic effort to identify the factors underlying adherence to COVID-19 social distancing messages. Everett et al. (2020) revealed that highlighting individuals' responsibilities to one another increased the effectiveness of a message. Barari et al. (2020) argued that messages were more effective when enriched with suggestions on how to make self-isolation easier. Pfattheicher et al. (2020) further showed that inducing empathy increased motivations to follow the messages. More relevant to our work, some researchers have explored the effects of using prosocial and self-interested appeals in a message. Heffner et al. (2020) compared messages that used fear (e.g., millions of people will die) with prosocial appeals (e.g., everyone's actions help society) and showed that prosocial messages were more likely to induce emotional responses and compliance. Jordan et al. (2020) compared messages that implied a threat to the individual (e.g., “don't get it”), a threat to the public (e.g., “don't spread it”), and both (e.g., “don't get and spread it”) and revealed that messages that implied a threat to the public were more effective in the early periods of the pandemic. By contrast, Falco and Zaccagni (2020) showed that reminders that emphasized the consequences of non-compliance for the individual or her family (vs. unknown others or the country's healthcare system) were more effective in motivating compliance. Raihani and de-Wit (2020) further showed that subjective concern in terms of the self and one's family was a stronger predictor of preventive behavior than more broadly framed concern in terms of society. Although common sense would suggest that the average person would react with self-interested motives at the expense of others in such a large-scale emergency response situation, these articles present mixed findings. Extending this emerging line of work, we explore novel variables that have not been examined in the COVID-19 context: (1) the effect of using prosocial vs. self-interested appeals on the persuasiveness of a social distancing message by testing different pronoun usage (“our” vs. “your”) in the message, (2) motivating willingness to help others as an outcome of using prosocial vs. self-interested appeals in a social distancing message, (3) the moderating roles of fear of coronavirus and locus of control on the proposed effects, and (4) the mediating role of feeling joint responsibility to protect from coronavirus behind the moderated effect of message type (prosocial vs. self-interested). To discuss our hypotheses, we next turn to the literature on prosocial vs. self-interest motivations.

According to the traditional economic view, individuals are self-interested; they act with the aim of maximizing their own utility. Yet, for decades, research has presented that people do not always act in their sole interests; they are often motivated by prosocial motives and act for the well-being of others (Comte, 1875). Predominant evidence suggests that self-interested appeals in a communication message primarily help fulfill egoistic motives of the target audience (Cialdini et al., 1997), and prosocial appeals help fulfill altruistic motives (Batson, 1990). Concerning public health messages, research shows that both personal and social benefit appeals may encourage preventive behaviors. On the one hand, prosocial appeals are shown to be more effective in motivating vaccination intentions against diseases (e.g., Kelly and Hornik, 2016; Li et al., 2016; Betsch et al., 2017) and encouraging hand washing to protect others in society (Grant and Hofmann, 2011). On the other hand, self-interested appeals are shown to be more influential for individuals who are highly concerned about a disease (Chang, 2011) and are at high risk of getting it (Vietri et al., 2012). Some other studies failed to find a difference between the effectiveness of two motives (e.g., Gerend and Barley, 2009; Hendrix et al., 2014).

Compared to previous pandemics, such as severe acute respiratory syndrome, Middle East respiratory syndrome, Ebola, and the Spanish flu outbreaks, COVID-19 spreads more quickly and easily through communities (Phillips, 2020; Woodley, 2020). It is also more difficult to trace COVID-19 because of the existence of mild or asymptomatic infections among the public. As often highlighted in official speeches (WHO, 2020a), it is of utmost importance for all individuals in society to act in solidarity in fighting against the pandemic. Adherence to social distancing regulations has become the acceptable behavior, hence practically the social norm in society (Cialdini et al., 1991) to curb the spread of the disease. In other words, complying with the recommendations is not only an individual but also a social decision. Based on these specific attributes of COVID-19, we argue that framing health messages around prosocial appeals and highlighting concern for everyone in the community would be more effective than framing messages around self-interested appeals by highlighting concern for the self only. More formally, we hypothesize that:

H1a: Prosocial (vs. self-interested) appeals will increase how persuasive individuals find the message to engage in social distancing.

As it brings a grim restriction to freedom, people may have difficulty understanding the importance of social distancing for different reasons. While some people question how one's behavior may hurt others' health concerning an invisible disease, others believe that the virus is unlikely to affect them (Springer, 2020). This suggests that the degree of fear toward the pandemic varies from person to person. Ahorsu et al. (2020) developed the fear of COVID-19 scale to capture this individual difference. High COVID-19 fear leads to intense emotional and physical consequences such as worry, anxiety, depression, and loss of sleep. Because individuals with a severe fear of coronavirus are generally more concerned about the negative consequences of the pandemic and are preoccupied with their well-being, they should have a greater base motivation to take actions to protect themselves from the disease. This, predictably, will make them less attentive to the differences in the framing of a social distancing message compared to people with lower levels of fear of coronavirus. Consequently, people with lower (vs. higher) coronavirus fear will be more attentive to subtle changes in the message framing and find it more persuasive when the message is framed around benefits for the whole society (vs. the self). Formally stated, we hypothesize that:

H1b: Prosocial (vs. self-interested) appeals will increase how persuasive individuals find the message to engage in social distancing more among individuals with lower levels of fear of coronavirus than individuals with higher levels of fear of coronavirus.

The degree of adherence to preventive measures may also depend on individuals' perceived sense of control. According to Rotter (1966), people differ in the perceived level of control that they have over situations and experiences that affect their lives. Some people believe to have a higher sense of control over what happens around them. This chronic sense of control is indicated as “locus of control” and is measured with a unidimensional scale (Chaxel, 2016). People who are at the higher (vs. lower) end of this scale are likely to believe that they can (vs. cannot) control the outcomes of events that take place in their surroundings (Burroughs and Glen Mick, 2004; Chaxel, 2016). Motivated by this, we predict that the locus of control will influence how individuals evaluate a social distancing message. We expect that people with higher levels of locus of control will believe that they can protect themselves from COVID-19 by taking necessary precautions and will be less influenced by a social distancing message. However, people with lower levels of locus of control will be more influenced by external warnings and hence find social distancing messages framed around prosocial (vs. self-interested) appeals more persuasive compared to those with higher levels of locus of control. Therefore, we hypothesize that:

H1c: Prosocial (vs. self-interested) appeals will increase how persuasive individuals find the message to engage in social distancing more among individuals with lower levels of locus of control than individuals with higher levels of locus of control.

COVID-19 has threatened lives in many ways. With lots of people suffering from social, economic, physical, and mental problems, community support has become especially important in coping with the adverse effects of the pandemic. In times of social distancing, people can help those in need through several means such as by sharing one's resources or donating money. Although identifying the factors that influence citizens' helping inclinations during the pandemic is crucial, it is an underresearched topic. Most relevant to our work, prior research has explored whether prosocial and self-interested motives drive helping behavior in diverse domains such as charitable donation (e.g., Brunel and Nelson, 2000; Schlosser and Levy, 2016), proenvironmental behavior (e.g., Griskevicius et al., 2010), organ donation (e.g., Pessemier et al., 1977), and volunteerism (e.g., Mowen and Sujan, 2005), as well as in for-profit organizations contexts (e.g., Ryoo et al., 2020). This research stream provides supporting evidence for both views that people may help others for personal benefits or for the good of society at large. We predict that if a social distancing message emphasizes everyone's well-being in society, as opposed to an individual's own well-being, it will increase people's concern for each other and their willingness to engage in helping behavior. Supporting this argument, research on self-construals reveals that priming the self as a socially embedded entity connected to others (i.e., interdependent self-construal) rather than as an autonomous entity distinct from others (i.e., independent self-construal) can motivate prosocial behavior. For example, activating interdependent self-construal promotes valuing collectivistic goals and perceiving higher obligations toward others in one's social network (Gardner et al., 1999) and motivates collaboration with others in sharing environmental resources (Arnocky et al., 2007). While we do not prime self-construals in this research, these findings support our view that highlighting concern for one's community at large, rather than the individual self, may motivate individuals more to help others during the pandemic. Summing up, we hypothesize that:

H2a: Prosocial (vs. self-interested) appeals in a social distancing message will motivate individuals more to help others.

People become fearful when they experience danger or threat in life (LaTour and Rotfeld, 1997), and this emotion intensifies in-group support as a coping mechanism (Fritsche et al., 2008). Based on this, we think that the level of fear of coronavirus may affect individuals' willingness to help each other during the pandemic. Individuals with higher levels of coronavirus fear tend to pay more attention to the frightening aspects of the disease and take it more seriously as they see it as a threat to their lives (Ahorsu et al., 2020). Accordingly, they are more effortful in combatting the disease compared to people with lower levels of fear (Harper et al., 2020). We predict that the higher salience of and concern about the pandemic will enhance the need for solidarity among people with higher fear and make them more considerate and empathic toward other individuals' needs. In other words, they will be more willing to help others regardless of being exposed to an external message. However, those with lower fear and concern about the disease will be more influenced by an external message in their motivations to help others. Therefore, we predict that prosocial (vs. self-interested) messages will be more persuasive in motivating willingness to help for those with lower levels of fear compared to those with higher levels of fear. More formally stated:

H2b: Prosocial (vs. self-interested) appeals in a social distancing message will motivate individuals with lower levels of fear of coronavirus more than individuals with higher levels of fear of coronavirus to help others.

The belief that one can create a difference in the sufferer's life by satisfying his/her needs is an important factor that affects the extent of willingness to help someone in need (Lerner and Reavy, 1975). Related to this, the locus of control influences not only how people respond to what happens in their surroundings but also their motivation to take action. People with higher (vs. lower) levels of locus of control have a higher belief that they can influence the lives of those who are in need and are more likely to engage in helping others with an internal motivation (Lerner and Reavy, 1975). Accordingly, we expect that people with a higher (vs. lower) locus of control will believe that they can play a role in improving others' well-being during a pandemic to a higher extent. They will be more likely to help others without necessarily being exposed to an external message. On the other hand, those with a lower locus of control will be more extrinsically motivated and hence will be more influenced by an external message in helping others. Therefore, we predict that prosocial (vs. self-interested) social distancing messages will be more persuasive in motivating willingness to help for those with lower levels of locus of control compared to those with higher levels of locus of control. More formally, we hypothesize that:

H2c: Prosocial (vs. self-interested) appeals in a social distancing message will motivate individuals with lower levels of locus of control more than individuals with higher levels of locus of control to help others.

One aim of our research is to understand the underlying reason for the higher effectiveness of prosocial (vs. self-interested) social distancing messages among people with lower coronavirus fear and lower locus of control. Extant research has mostly examined egoistic motivations (e.g., to reduce one's chances of getting a disease; Brunel and Nelson, 2000; or to improve one's current status; Schlosser and Levy, 2016) as the primary reason for the effectiveness of self-interested messages and altruistic motivations (e.g., to give back to the society; Schlosser and Levy, 2016; or to help make the world a better place for everyone, White and Peloza, 2009) for the effectiveness of prosocial messages. Because COVID-19 has a very high transmission rate and an unbalanced impact on individuals, we suggest a different motivation: the need for collective effort to combat the pandemic. The more people obey preventive measures, the higher the indirect protection is for others. Therefore, we predict that a social distancing message with prosocial (vs. self-interested) appeals will motivate people with lower (vs. higher) levels of coronavirus fear and locus of control to a higher extent to comply with the message and engage in helping behaviors, by inducing the feeling of having joint responsibility to protect from the disease. More formally stated:

H3a: The feeling of joint responsibility to protect from coronavirus will mediate the moderated effect of message type by coronavirus fear on how persuasive individuals find the message to engage in social distancing.H3b: The feeling of joint responsibility to protect from coronavirus will mediate the moderated effect of message type by coronavirus fear on how much the message motivates individuals to help others.H3c: The feeling of joint responsibility to protect from coronavirus will mediate the moderated effect of message type by the locus of control on how persuasive individuals find the message to engage in social distancing.H3d: The feeling of joint responsibility to protect from coronavirus will mediate the moderated effect of message type by the locus of control on how much the message motivates individuals to help others.

Next, we present three studies to test the hypotheses. We operationalize messages with prosocial and self-interested appeals by using different pronouns in the message. Specifically, we use the “our” pronoun to highlight that a social distancing message benefits everyone in the society and the “your” pronoun to highlight that the message benefits the individual only. It is important to note that while prosocial message appeals may refer to the benefits of one's actions for other individuals expressing altruistic values (e.g., “I want to help others”; Brunel and Nelson, 2000), they are also used to indicate the larger community that includes the message recipient as well (e.g., “I help to make the world a better place for everyone”; White and Peloza, 2009; Schlosser and Levy, 2016, “I have environmental concerns because of the consequences for all people/the people in my community”; Schultz, 2001; Schultz et al., 2005). In line with real-life COVID-19 social distancing messages, we follow the latter usage and imply “everyone in the community” in the prosocial message condition.

## Overview of Studies

Each of the three studies includes an experiment that was created by using Qualtrics online survey tool. In all studies, participation was voluntary; informed consent was obtained, and participants were assured that their responses would be kept confidential.

Studies use real-life “stay at home” and “social distancing” declarations that emphasize the importance of message compliance to protect from coronavirus. To increase the representatives of the samples, we employed varied participant groups with respect to their demographic and geographic characteristics. Specifically, study 1 employed student participants in exchange for partial course credit, and studies 2 and 3 recruited participants from a large online pool in return for a monetary reward.

We used SPSS 19 to analyze the data and SPSS PROCESS macro (version 3.14) for the moderation and mediation analyses. This macro was developed by Hayes (2013), and it conducts mediation analysis by using bootstrap methods. In each study, we used this method with 5,000 bootstraps to test the mediation hypothesis. The bootstrap method tests the significance of the indirect effect of the independent variable on the dependent variable through the mediator (Shrout and Bolger, 2002) and detects the existence of the mediating effect when the confidence interval (CI) for the indirect effect does not include zero (MacKinnon et al., 1995; Wang et al., 2017).

## Study 1

Study 1 explores the effect of using prosocial (vs. self-interested) appeals in social distancing messages on persuading individuals to comply with the message (H1a) and motivating them to help others (H2a).

### Participants, Design, and Procedure

This study was conducted with 119 students (50 men, 67 women, mean_age_ = 20.99 years, SD = 2.10 years; two people did not reveal their age and gender information) between April 22 and May 6, 2020, at a private university in Turkey. One hundred thirteen participants (48 men, 65 women, mean_age_ = 21.06 years, SD = 1.86 years) remained in the data after attention checks. The study was conducted in participants' native language to make the stimuli realistic and prevent any language-related barriers in collecting data.

To manipulate the message type, a one-way between-subjects design was used (message type: self-interested vs. prosocial). Participants were randomly assigned to the manipulated conditions in each study. Specifically, participants in the self-interested (prosocial) message condition were given a message that reads, “For your own (all our) health, stay at home.” A coronavirus illustration was included in the flyers to delineate the concept of the message (see Appendix for the stimuli). To make sure that participants read and processed the message, they were asked to write their thoughts in an open-ended format (Rucker et al., 2011). Then, participants indicated how persuasive they found the message, with two items (“How persuasive did you find this message to stay at home?” and “How convincing did you find this message to ensure self-isolation?”; 1–7 = “not at all” to “very much”). We took the average of these two items to create a composite score of the persuasiveness of the message to self-isolate [*r*(113) = 0.85, *p* < 0.0001].

Next, participants indicated how much the message motivated them to engage in the following acts (“To help those in need”; “To share resources with other people”; and “To donate money to those in need”; 1–7 = “not at all” to “very much”). We took the average of these three items to create a composite score of how much the message motivates individuals to help others (Cronbach α = 0.95).

Participants completed the study by providing their age and gender information.

### Results

#### Persuasiveness of the Message to Self-Isolate

We conducted a one-way analysis of variance (ANOVA) to test H1a. The results revealed that participants perceived the prosocial message (mean_prosocial_ = 4.54, SD = 1.34) as more convincing to self-isolate and stay at home than the self-interested message [mean_self−interested_ = 3.58, SD = 1.64; *F*_(1, 111)_ = 11.70, *p* = 0.001, $\eta_{\text{p}}^{2}$ = 0.10].

#### How Much the Message Motivates Individuals to Help Others

We conducted a one-way ANOVA to test H2a. The results revealed that participants in the prosocial message condition were more motivated to help others than those in the self-interested message condition [mean_prosocial_ = 4.25, SD = 1.74; mean_self−interested_ = 2.95, SD = 1.82; *F*_(1, 111)_ = 15.01, *p* < 0.0001, $\eta_{\text{p}}^{2}$ = 0.12].

The results thus support the hypotheses proposed for study 1. The results show that when social distancing messages use prosocial (vs. self-interested) appeals, people perceive the message as more persuasive to self-isolate. Also, prosocial (vs. self-interested) messages motivate people more to help others.

## Study 2

Study 2 tests the effect of message type on persuading individuals to engage in social distancing (H1a) and motivating individuals to help others (H2a) by using a different participant group to increase the external validity of the results. It also tests the role of coronavirus fear (H1b and H2b) and the mediating mechanism behind the proposed effects (H3a and H3b).

### Participants, Design, and Procedure

This study was conducted on Prolific Academic, a UK-based crowdsourcing platform for scientific research. We recruited 202 participants from the United States on May 26, 2020 (90 men, 111 women, mean_age_ = 36.94 years, SD = 13.08 years; one person did not reveal his/her gender and age information). Message type was manipulated as in study 1 with one difference. Messages were shown to participants in the form of a flier with identical people icons (one person in the self-interested message condition, four people in the prosocial message condition) rather than coronavirus illustrations. People icons were added to the flier to increase the strength of the manipulation. Seeing one (vs. multiple) person icon(s) in the self-interested (prosocial) message condition should ensure that “your own (all our)” pronoun is used to imply the message recipient's (everyone's) well-being.

After reading the message and writing their thoughts about it, participants indicated (1) how convincing they found the message to stay at home (1–7 = “not at all” to “very much”) and (2) how much the message motivated them to help others, with the three items used in study 1 (Cronbach α = 0.91). Then, participants responded to the following two items intended to understand the extent to which they thought that protection from coronavirus was everyone's joint responsibility (“To what extent did this message make you feel that protection from coronavirus is a common responsibility of all people?” and “To what extent did this message make you feel responsible for other people's well-being?”; 1–7 = “not at all” to “very much”). We took the average of these items to create a composite score of the feeling of joint responsibility to protect from coronavirus [*r*(202) = 0.82, *p* < 0.0001].

Afterward, participants responded to the fear of coronavirus scale (Ahorsu et al., 2020), which includes the following seven items (“I am most afraid of coronavirus”; “It makes me uncomfortable to think about coronavirus”; “My hands become clammy when I think about coronavirus”; “I am afraid of losing my life because of coronavirus”; “When watching news and stories about coronavirus on social media, I become nervous”; “I cannot sleep because I'm worrying about getting coronavirus”; and “My heart races or palpitates when I think about getting coronavirus”; 1–7 = “not at all” to “very much”). We took the average of these items to create a composite score of fear of coronavirus (Cronbach α = 0.90).

Participants completed the study by providing their age and gender information.

### Results

#### Persuasiveness of the Message to Stay at Home

We used PROCESS macro (model 1) to test H1a and H1b. In this and the following study, the self-interested (prosocial) message condition was coded as zero (one). The effect of message type was positive (β = 1.33, SE = 0.56, *t* = 2.37, *p* = 0.02). The prosocial message thus was found more effective in convincing participants to stay at home. The effect of fear of coronavirus was also positive (β = 0.41, SE = 0.12, *t* = 3.39, *p* < 0.001); this shows that when the fear increases, social distancing messages, regardless of their appeal, are perceived as more convincing.

**Figure 1:**
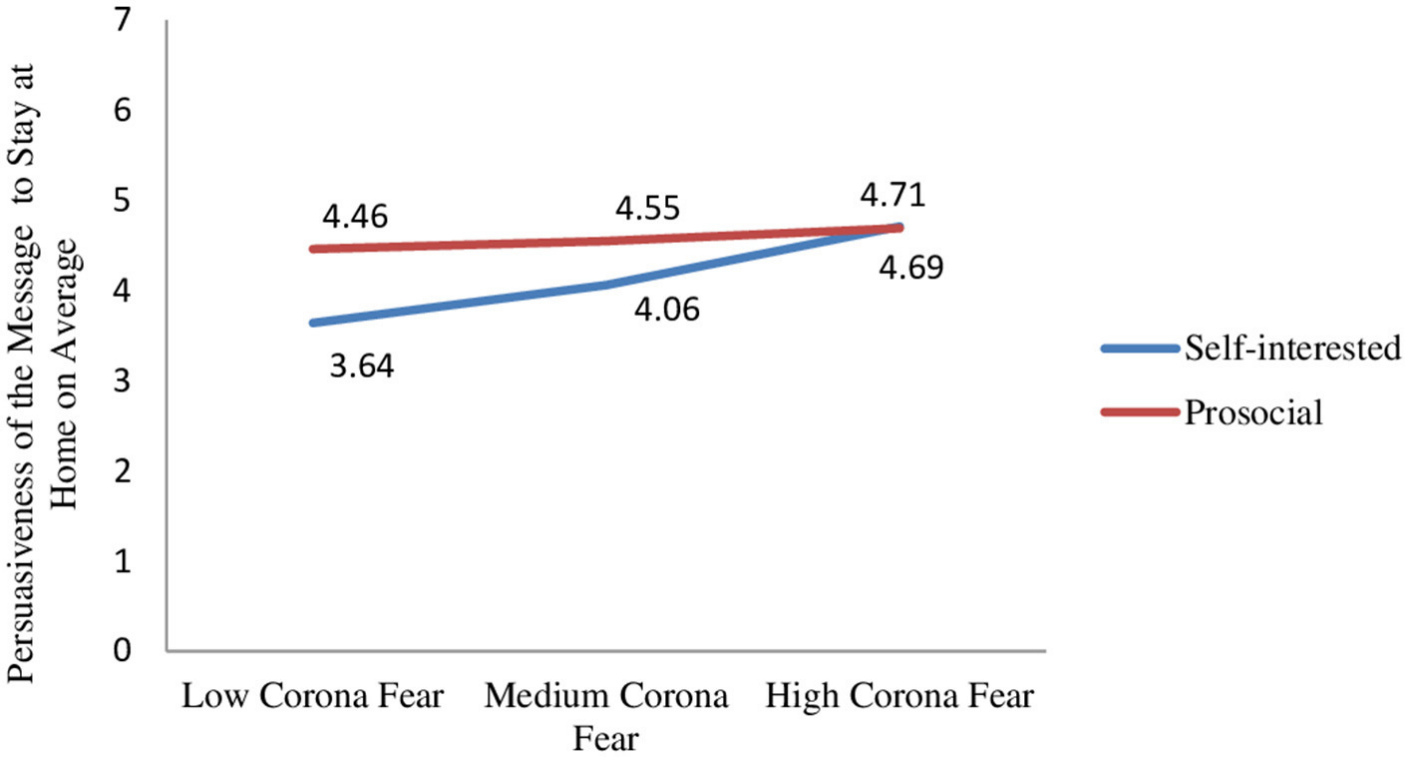
Effect of message type x fear of coronavirus on persuasiveness of the message to stay at home.

Also, the two-way interaction between message type and fear of coronavirus was marginally significant (β = −0.33, SE = 0.18, *t* = −1.82, *p* = 0.07). Participants with low and medium levels of coronavirus fear perceived the prosocial (vs. self-interested) message as more convincing to stay at home (β_low_fear_ = 0.82, SE_low_fear_ = 0.33, *t* = 2.50, *p* = 0.01; β_medium_fear_ = 0.50, SE_medium_fear_ = 0.24, *t* = 2.06, *p* = 0.04). However, participants with high levels of coronavirus fear found the messages equally effective (β_high_fear_ = −0.02, SE_high_fear_ = 0.33, *t* = −0.05, *p* = 0.96; Figure 1).

#### How Much the Message Motivates Individuals to Help Others

We used PROCESS macro (model 1) to test H2a and H2b. The effect of message type was positive (β = 2.21, SE = 0.51, *t* = 4.37, *p* < 0.0001). The prosocial message thus motivated participants more to help others. Additionally, the effect of fear of coronavirus was positive (β = 0.56, SE = 0.11, *t* = 5.09, *p* < 0.0001); this indicates that when coronavirus fear increases, social distancing messages, regardless of their appeal, motivate individuals more to help others.

**Figure 2:**
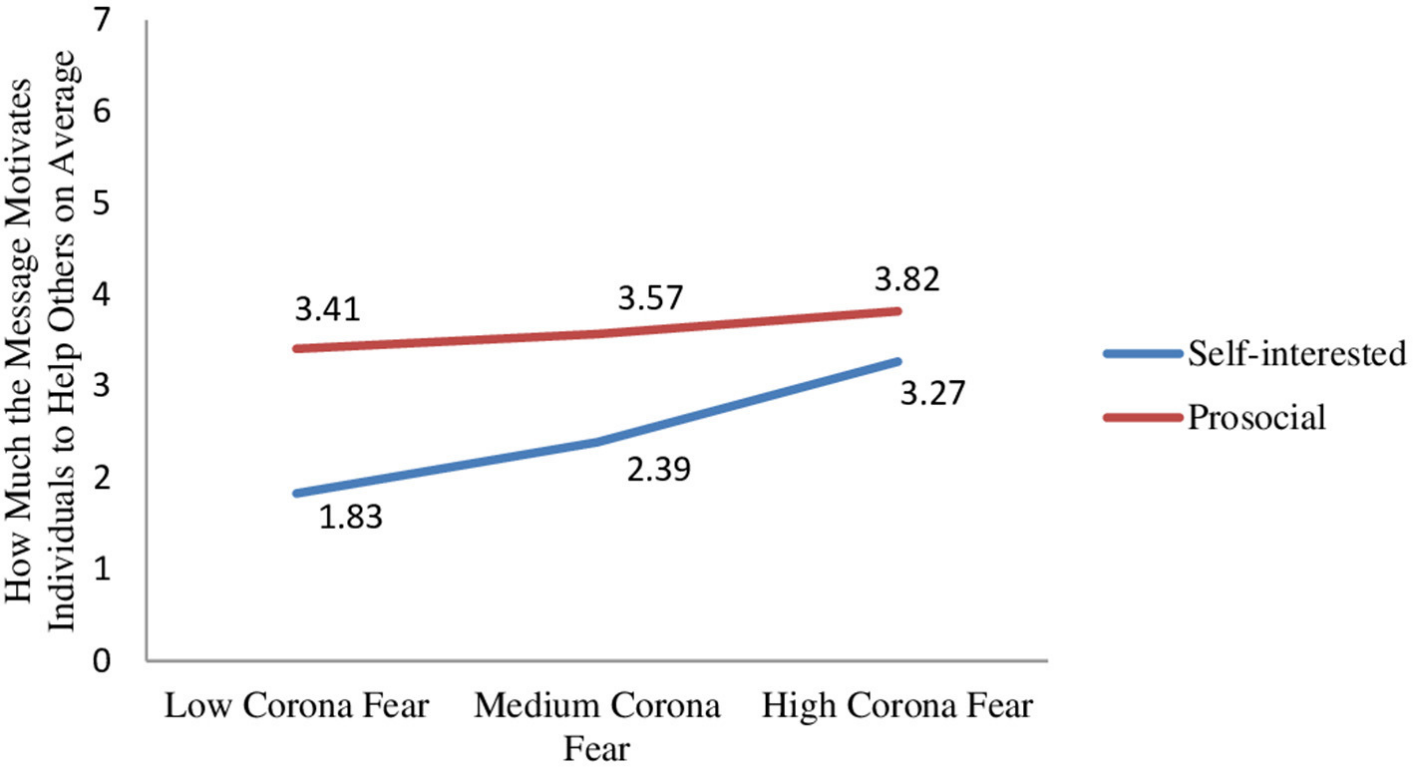
Effect of message type x fear of coronavirus on how much the message motivates individuals to help others.

Importantly, the two-way interaction between message type and fear of coronavirus was significant (β = −0.40, SE = 0.16, *t* = −2.49, *p* = 0.01). The prosocial message motivated participants with low and medium (high) levels of coronavirus fear to help others significantly (marginally) more than the self-interested message (β_low_fear_ = 1.58, SE_low_fear_ = 0.30, *t* = 5.33, *p* < 0.0001; β_medium_fear_ = 1.18, SE_medium_fear_ = 0.22, *t* = 5.44, *p* < 0.0001; β_high_fear_ = 0.55, SE_high_fear_ = 0.30, *t* = 1.85, *p* = 0.07; Figure 2).

#### Feeling Joint Responsibility to Protect From Coronavirus as the Mediator

We used PROCESS macro (model 1) to test how the message type, fear of coronavirus, and their interaction affect the mediator. The results revealed that the prosocial message created a higher feeling of joint responsibility to protect from coronavirus than the self-interested message (β = 2.23, SE = 0.60, *t* = 3.73, *p* < 0.001). The fear of coronavirus also had a positive effect on the mediator (β = 0.40, SE = 0.13, *t* = 3.09, *p* = 0.002). However, the two-way interaction between message type and fear of coronavirus was non-significant (β = −0.21, SE = 0.19, *t* = −1.12, *p* = 0.26).

#### Mediation Analysis for Persuasiveness of the Message to Stay at Home

We used PROCESS macro (model 5) to test H3a. The results revealed that feeling joint responsibility to protect from coronavirus had a significant effect on persuasiveness of the message to stay at home (β = 0.55, SE = 0.05, *t* = 10.17, *p* < 0.0001; see Table 1 for the regression analysis), and it is the proposed mediating factor, as the 95% CI for the indirect effect excluded zero (β = 0.84, SE = 0.16, CI = 0.53–1.17).

**Table 1:** Regression results for the mediation analysis on persuasiveness of the message to stay at home in study 2.

**Effect**	**β**	**SE**	***t***
Direct effect of X on Y	0.10	0.47	0.21
Direct effect of Mo on Y	0.19	0.10	1.90^*^
Direct effect of X × Mo on Y	−0.21	0.15	−1.43
Direct effect of Me on Y	0.55	0.05	10.17^**^

*X is the message type (i.e., the independent variable), Mo is fear of coronavirus (i.e., the moderator), Me is feeling joint responsibility to protect from coronavirus (i.e., the mediator), and Y is persuasiveness of the message to stay at home (i.e., the dependent variable). X × Mo is the moderated effect of message type on Y*.

* *p < 0.1*,

** *p < 0.01*.

#### Mediation Analysis for How Much the Message Motivates Individuals to Help Others

We used PROCESS macro (model 5) to test H3b. The results revealed that feeling joint responsibility to protect from coronavirus had a significant effect on how much the message motivates individuals to help others (β = 0.39, SE = 0.05, *t* = 7.24, *p* < 0.0001; see Table 2 for the regression analysis), and it is the proposed mediating factor, as the 95% CI for the indirect effect excluded zero (β = 0.59, SE = 0.14, CI = 0.34–0.88).

**Table 2:** Regression results for the mediation analysis on how much the message motivates individuals to help others in study 2.

**Effect**	**β**	**SE**	***t***
Direct effect of X on Y	1.34	0.47	2.88^**^
Direct effect of Mo on Y	0.40	0.10	4.03^**^
Direct effect of X × Mo on Y	−0.32	0.14	−2.22^*^
Direct effect of Me on Y	0.39	0.05	7.24^**^

*X is the message type (i.e., the independent variable), Mo is fear of coronavirus (i.e., the moderator), Me is feeling joint responsibility to protect from coronavirus (i.e., the mediator), and Y is how much the message motivates individuals to help others (i.e., the dependent variable). X × Mo is the moderated effect of message type on Y*.

* *p < 0.05*,

** *p < 0.01*.

In summary, the results support the hypotheses proposed for study 2. The results show that social distancing messages with prosocial (vs. self-interested) appeals are more effective in driving message compliance and helping behavior. Moreover, the effectiveness of the prosocial message is moderated by fear of coronavirus. We predict that people with high levels of coronavirus fear are more concerned about the negative effects of the pandemic; hence, their base motivation to take precautions against COVID-19 and to help others is higher. Accordingly, the results show that people with high levels of coronavirus fear find the two messages equally persuasive to comply with social distancing; however, people with low and medium levels of coronavirus fear are more convinced to comply with social distancing messages that use prosocial (vs. self-interested) appeals. Furthermore, our results show that prosocial (vs. self-interested) messages motivate individuals with low and medium levels of fear to help others more than those with high levels of fear. These moderated effects of message type occur because of feeling a collective responsibility in protecting from the disease.

These results replicate the findings of study 1 by using a different participant group and hence increase the generalizability of the results. This study recruited participants from a Western culture (United States), whereas study 1 had student participants from an Eastern culture (Turkey). Demonstrating that social distancing messages with prosocial (vs. self-interested) appeals are more persuasive in driving compliance and motivating helping behavior in both studies provides evidence that the results are robust across different cultures.

## Study 3

Study 3 aims to show that the locus of control creates a boundary condition for the persuasiveness of different message appeals in driving message compliance (H1c) and motivating helping behavior (H2c). Additionally, this study investigates the mediating mechanism behind the moderated effect of message type by the locus of control on persuasiveness of the message to keep a physical distance with others (H3c) and helping others in need (H3d).

### Participants, Design, and Procedure

This study was conducted on Prolific Academic on August 20, 2020. Two hundred one people participated from the United States (113 women, 88 men, mean_age_ = 35.58 years, SD = 12.48 years).

As in previous studies, we used a one-way between-subjects design (message type: self-interested vs. prosocial). We slightly changed the message type manipulation to increase the generalizability of the results. Specifically, participants in the self-interested (prosocial) message condition were given a message that reads, “For your own (all our) health, keep your physical distance with others.” WHO (2020b) characterized COVID-19 as a pandemic on March 11, 2020. Shortly after this announcement, many countries around the world declared strict stay-at-home orders. Thus, in studies 1 and 2, which were conducted in April and May, respectively, we used the “stay at home” phrase in the messages. By midsummer, many countries eased restrictions by replacing stay-at-home warnings with social distancing recommendations. Because study 3 was conducted in August 2020, we used the more realistic “Keep your physical distance” phrase in the message flier. Also, we used coronavirus illustrations as in study 1 (rather than people icons as in study 2) for a more stringent manipulation of message type and to increase the robustness of the results.

Unlike previous studies, participants did not provide their thoughts about the message, but directly indicated the extent to which the message convinced them to keep a physical distance with two items (“How motivating did you find this message in keeping a physical distance with others?” and “How persuasive did you find this message in taking precautions against COVID-19, such as wearing masks and social distancing?”; 1–7 = “not at all” to “very much”). We took the average of these two items to create a composite score of the persuasiveness of the message to keep a physical distance [*r*(201) = 0.82, *p* < 0.0001].

Participants then indicated how much the message motivated them to help others, with the same three items that were used in previous studies (Cronbach α = 0.91). They also reported how much the message induced the feeling of joint responsibility to protect from coronavirus, with three items. In addition to the two items that were used in study 2, an additional item was used to further delineate the importance of collective effort in protecting from coronavirus: “To what extent did this message make you feel that protection from COVID-19 is only possible with collective effort of everyone?” (1–7 = “not at all” to “very much”). The three items were averaged to create a composite score of feeling joint responsibility to protect from coronavirus (Cronbach α = 0.92).

Next, participants reported their locus of control by responding to six items adapted from Rotter (1966) locus of control scale (“People's misfortunes result from the mistakes they make”; “When I make plans, I am almost certain that I can make them work”; “By taking an active part in political and social affairs the people can control world events”; “It is impossible for me to believe that chance or luck plays an important role in my life”; “Most misfortunes are the result of lack of ability, ignorance, laziness, or all three”; and “What happens to me is my own doing”; 1–7 = “not at all” to “very much”). We took the average of these six items to create a composite score of locus of control (Cronbach α = 0.72).

Finally, participants reported their age and gender information.

### Results

#### Persuasiveness of the Message to Keep a Physical Distance With Others

We used PROCESS macro (model 1) to test H1a and H1c. The results showed a positive effect of message type (β = 2.36, SE = 0.97, *t* = 2.42, *p* = 0.02). Thus, the prosocial message was found more persuasive to keep a physical distance with others. The effect of locus of control was also significant (β = 0.62, SE = 0.18, *t* = 3.43, *p* < 0.001), showing that when locus of control increases, persuasiveness of social distancing messages, regardless of message appeal, increases as well.

**Figure 3:**
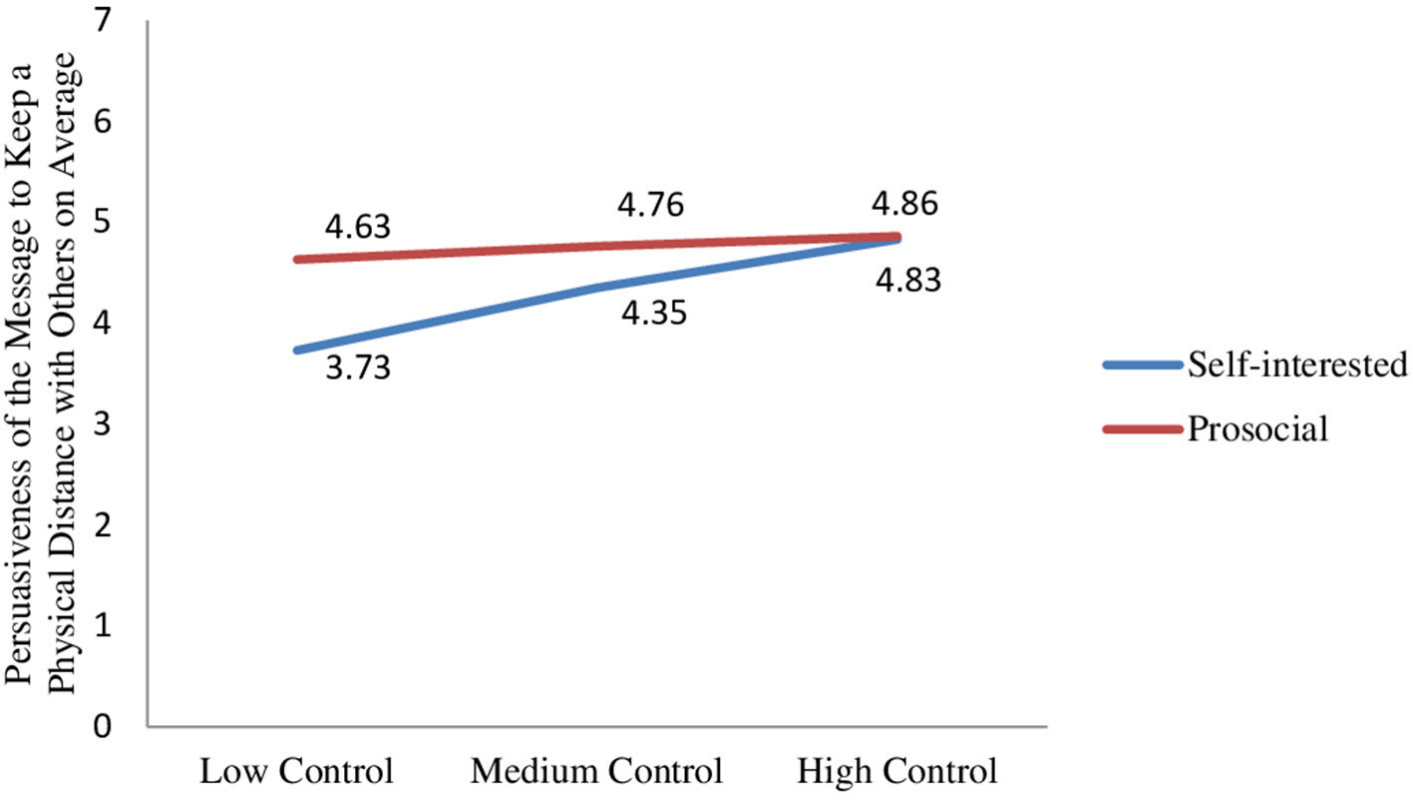
Effect of message type x locus of control on persuasiveness of the message to keep a physical distance with others.

Importantly, the two-way interaction between message type and locus of control was significant (β = −0.49, SE = 0.24, *t* = −2.02, *p* = 0.04). Participants with low (medium) levels of locus of control found the prosocial message significantly (marginally) more persuasive to keep a physical distance with others than the self-interested message (β_low_control_ = 0.90, SE_low_control_ = 0.32, *t* = 2.80, *p* = 0.01; β_medium_control_ = 0.41, SE_medium_control_ = 0.23, *t* = 1.80, *p* = 0.07). However, participants with high levels of locus of control perceived the two messages as equally effective (β_high_control_ = 0.03, SE_high_control_ = 0.30, *t* = 0.11, *p* = 0.91; Figure 3).

#### How Much the Message Motivates Individuals to Help Others

We used PROCESS macro (model 1) to test H2a and H2c. The effect of message type was positive (β = 3.45, SE = 1.03, *t* = 3.42, *p* = 0.001), showing that the message with prosocial appeal motivated participants more to help others. The effect of locus of control was also positive (β = 0.88, SE = 0.19, *t* = 4.61, *p* < 0.0001). Thus, when the locus of control increases, social distancing messages, regardless of their appeal, motivate people more to help others.

**Figure 4:**
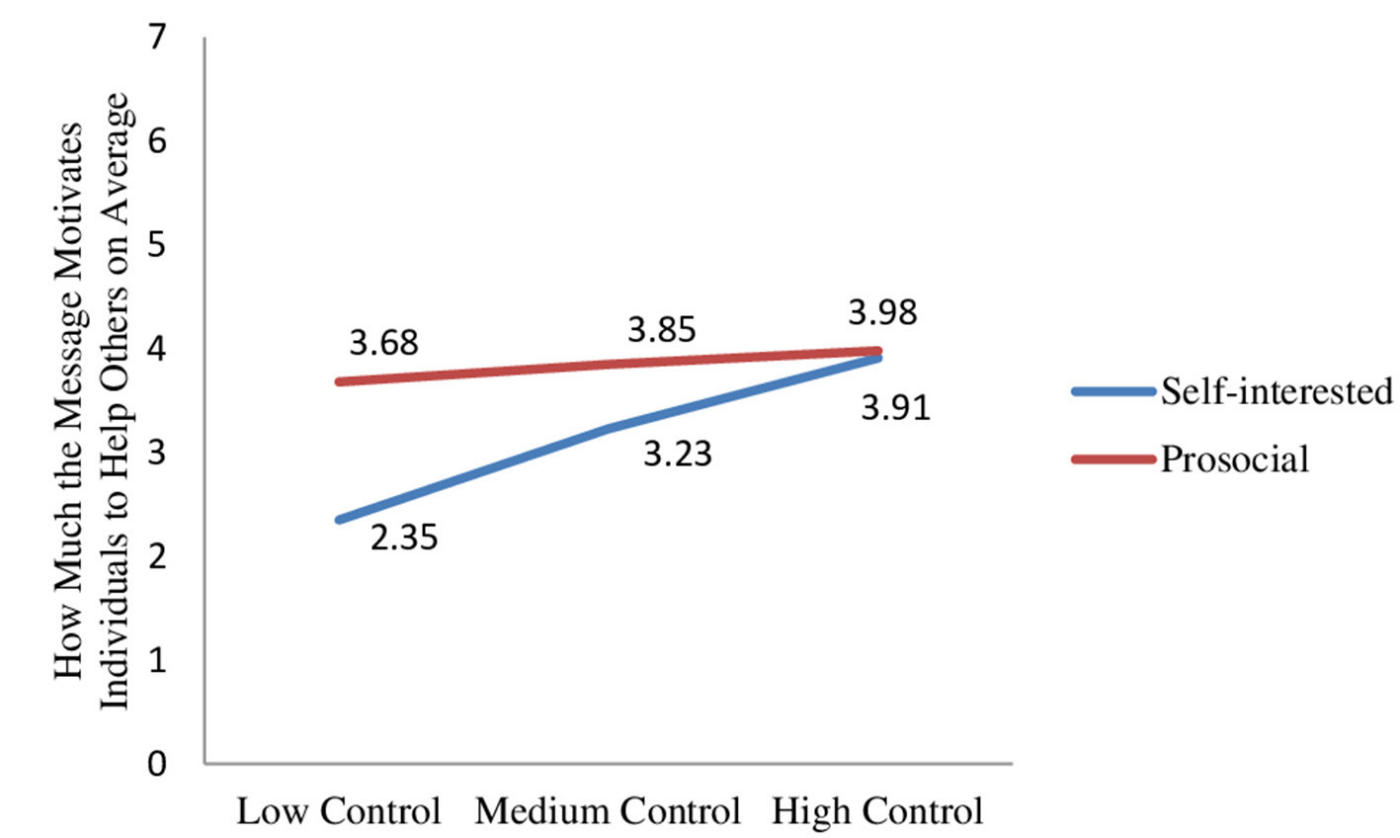
Effect of message type x locus of control on how much the message motivates individuals to help others.

Furthermore, the two-way interaction between the message type and the locus of control was significant (β = −0.71, SE = 0.26, *t* = −2.78, *p* = 0.01). The prosocial message motivated participants with low and medium levels of locus of control to help others more than the self-interested message did (β_low_control_ = 1.33, SE_low_control_ = 0.34, *t* = 3.90, *p* < 0.001; β_medium_control_ = 0.62, SE_medium_control_ = 0.24, *t* = 2.56, *p* = 0.01). However, the messages were equally effective in motivating helping behavior for participants with high levels of locus of control (β_high_control_ = 0.07, SE_high_control_ = 0.32, *t* = 0.21, *p* = 0.84; Figure 4).

#### Feeling Joint Responsibility to Protect From Coronavirus as the Mediator

We used PROCESS macro (model 1) to test how the message type, locus of control, and their interaction affect the mediator. The results revealed that the prosocial message created a higher feeling of joint responsibility to protect from coronavirus than the self-interested message (β = 3.92, SE = 0.99, *t* = 3.95, *p* < 0.001). Also, locus of control had a positive effect on the mediator (β = 0.73, SE = 0.18, *t* = 4.00, *p* < 0.001). Importantly, the two-way interaction between message type and locus of control was significant (β = −0.62, SE = 0.24, *t* = −2.55, *p* = 0.01).

#### Mediation Analysis for Persuasiveness of the Message to Keep a Physical Distance With Others

To test H3c, we used PROCESS macro (model 8). The results revealed that feeling joint responsibility to protect from coronavirus had a significant effect on persuasiveness of the message to keep a physical distance with others (β = 0.63, SE = 0.05, *t* = 11.82, *p* < 0.0001; see Table 3 for the regression analysis), and it is the proposed mediating factor, as the 95% CI for the index of moderated mediation excluded zero (index = −0.40, SE = 0.15, CI = −0.70 to −0.10).

**Table 3:** Regression results for the mediation analysis on persuasiveness of the message to keep a physical distance with others in study 3.

**Effect**	**β**	**SE**	***t***
Direct effect of X on Y	−0.13	0.78	−0.17
Direct effect of Mo on Y	0.15	0.14	1.08
Direct effect of X × Mo on Y	−0.09	0.19	−0.48
Direct effect of Me on Y	0.63	0.05	11.82^*^

*X is the message type (i.e., the independent variable), Mo is locus of control (i.e., the moderator), Me is feeling joint responsibility to protect from coronavirus (i.e., the mediator), and Y is persuasiveness of the message to keep a physical distance with others (i.e., the dependent variable). X × Mo is the moderated effect of message type on Y*.

* *p < 0.01*.

#### Mediation Analysis for How Much the Message Motivates Individuals to Help Others

To test H3d, we used PROCESS macro (model 8). The results revealed that feeling joint responsibility to protect from coronavirus had a significant effect on how much the message motivates individuals to help others (β = 0.52, SE = 0.06, *t* = 8.05, *p* < 0.0001; see Table 4 for the regression analysis), and it is the proposed mediating factor, as the 95% CI for the index of moderated mediation excluded zero (index = −0.32, SE = 0.13, CI = −0.60 to −0.08).

**Table 4:** Regression results for the mediation analysis on how much the message motivates individuals to help others in study 3.

**Effect**	**β**	**SE**	***t***
Direct effect of X on Y	1.42	0.93	1.52
Direct effect of Mo on Y	0.50	0.17	2.89^**^
Direct effect of X × Mo on Y	−0.38	0.23	−1.71^*^
Direct effect of Me on Y	0.52	0.06	8.05^**^

*X is the message type (i.e., the independent variable), Mo is locus of control (i.e., the moderator), Me is feeling joint responsibility to protect from coronavirus (i.e., the mediator), and Y is how much the message motivates individuals to help others (i.e., the dependent variable). X × Mo is the moderated effect of message type on Y*.

* *p < 0.1*,

** *p < 0.01*.

Study 3 demonstrates that the locus of control moderates the effect of message type on how persuasive individuals find a social distancing message to keep a physical distance with others and also to help others. Because people with high levels of locus of control believe that they are able to control what happens around them, they are more likely to be intrinsically motivated to take actions to combat the disease. Consequently, the results demonstrate that people with high levels of locus of control do not react differently to social distancing messages with different appeals in terms of message compliance and helping others. However, social distancing messages framed around prosocial (vs. self-interested) appeals are more persuasive to drive compliance and motivate helping behavior among people with low and medium levels of locus of control as prosocial messages increase their feelings of having joint responsibility to protect from the pandemic. The results thus support the hypotheses proposed for study 3.

## General Discussion

Maintaining physical distance and direct contact among individuals has been the key means for preventing the spread of the devastating COVID-19 outbreak. As the mounting academic work reflects, government entities, businesses, non-governmental organizations, and health authorities must join forces to convey the importance of keeping physical distance to citizens around the globe. Given that it may take up to a few years until a vaccine is fully distributed and administered (Lurie et al., 2020), social distancing will maintain its position as one of the most important control mechanisms during the pandemic.

Accordingly, we are responding to urgent calls to find the most effective ways to convey social distancing messages to the public. By using realistic messages at different phases of the pandemic (beginning and midsummer), we explore the effect of using prosocial vs. self-interested appeals on evaluations of the message in two substantial domains: message compliance and helping behavior.

This research specifically contributes to the academic work on prosocial vs. self-interest motivations, message compliance, and helping behavior. Our theoretical contributions can be summarized as follows: (1) an increasing amount of academic work is undertaken to explore the factors that influence the effectiveness of social distancing messages. It is still not clear whether it is better to make personal benefits or social benefits central in developing social distancing messages. Our results suggest that prosocial messages that emphasize benefits for everyone in society are more effective than self-interested messages that emphasize benefits for the individual, in driving message compliance. (2) Identifying the factors that motivate individuals' helping behavior is especially important during the pandemic as many people are in dire need of community support. To the best of our knowledge, this is the first study that explores how preventive social distancing messages can indirectly influence citizens' helping behavior toward each other. Specifically, we demonstrate that social distancing messages with prosocial appeals can motivate helping behavior (such as by sharing one's resources and donating money) more than those with self-interested appeals. (3) We explore fear of coronavirus as a moderating variable in how individuals evaluate social distancing messages. We demonstrate that individuals with low and medium levels of coronavirus fear are more influenced by prosocial (vs. self-interested) messages in following social distancing recommendations and also in helping others. However, people with high levels of fear do not react differently to messages with different appeals in terms of message compliance. Also, our results show that prosocial (vs. self-interested) messages motivate individuals with low and medium levels of fear to help others more than those with high levels of fear. (4) We explore the locus of control as another moderating variable. Our findings show that individuals with low and medium levels of locus of control are more influenced by prosocial (vs. self-interested) messages in following social distancing recommendations and also in helping others. However, people with high levels of locus of control do not react differently to different message appeals in terms of message compliance or helping behavior. (5) Finally, we investigate why creating prosocial (vs. self-interested) messages is more persuasive in motivating compliance and helping behavior among people with low and medium levels of fear of coronavirus and locus of control. Because fighting against the COVID-19 pandemic requires social solidarity, the moderated effect of message type on message compliance and helping others occurs through the feeling of joint responsibility to protect from coronavirus (see Table 5 for a summary of all findings).

**Table 5:** Summary of all findings in studies 2 and 3.

**Study 2**		**Message type**	**Low coronavirus fear**	**Medium coronavirus fear**	**High coronavirus fear**	**Confidence interval for the mediation analysis**
H1a, H1b, and H3a are supported	Message compliance DV	β = 1.33, SE = 0.56, *t* = 2.37^**^	β = 0.82, SE = 0.33, *t* = 2.50^**^	β = 0.50, SE = 0.24, *t* = 2.06^**^	β = −0.02, SE = 0.33, *t* = −0.05	0.53 to 1.17
H2a, H2b, and H3b are supported	Helping DV	β = 2.21, SE = 0.51, *t* = 4.37^***^	β = 1.58, SE = 0.30, *t* = 5.33^***^	β = 1.18, SE = 0.22, *t* = 5.44^***^	β = 0.55, SE = 0.30, *t* = 1.85^*^	0.34 to 0.88
**Study 3**		**Message type**	**Low locus of control**	**Medium locus of control**	**High locus of control**	**Confidence interval for the mediation analysis**
H1a, H1c, and H3c are supported	Message compliance DV	β = 2.36, SE = 0.97, *t* = 2.42^**^	β = 0.90, SE = 0.32, *t* = 2.80^**^	β = 0.41, SE = 0.23, *t* = 1.80^*^	β = 0.03, SE = 0.30, *t* = 0.11	−0.70 to −0.10
H2a, H2c, and H3d are supported	Helping DV	β = 3.45, SE = 1.03, *t* = 3.42^***^	β = 1.33, SE = 0.34, *t* = 3.90^***^	β = 0.62, SE = 0.24, *t* = 2.56^**^	β = 0.07, SE = 0.32, *t* = 0.21	−0.60 to −0.08

* *p < 0.1*,

** *p < 0.05*,

*** *p < 0.01*.

Our findings provide clear implications for public policymakers, managers, and communication experts. Policymakers often ask whether a communication message should speak to the individual or the larger community to maximize the persuasive impact of a message. Social distancing was one of the key preventive measures in many past disease epidemics as well, such as the Spanish flu pandemic (Glass et al., 2006). Hence, such control policies may be in place during other contagious diseases we might face in the future. This necessitates policymakers to be more prepared in responding to these contagious diseases. Because of the ease of implementation of the language used in public health messages, our findings provide solid and quickly implementable suggestions on how to increase the persuasiveness of social distancing messages. Moreover, the literature on prosocial behavior shows that helping others increases the recipient's and the giver's well-being and happiness (e.g., Anik et al., 2009; Rudd et al., 2014). From the perspective of policymakers, framing the publicly conveyed social distancing messages around social benefits by slightly changing the pronouns used in the message can motivate individuals' willingness to help one another, and as a result, may contribute to the society's overall well-being.

Our moderation analyses show that social distancing messages do not influence everyone in the same way. People with low and medium levels of coronavirus fear and locus of control are shown to be more attentive to and influenced by prosocial messages than self-interested messages. On the other hand, people with high levels of coronavirus fear and locus of control are more inclined to take precautionary measures intrinsically, at the base level. Based on this, messages may be tailored differently in geographic locations where the number of cases is relatively low, and presumably, so is the level of fear in society. Moreover, prosocial messages that are designed for places with a lower number of cases may highlight one's ability to control his/her situation by taking action against the disease. For example, a message that emphasizes the uncontrollable transmission pace of COVID-19 may induce fear. The same message may highlight how one is able to control the transmission of the disease by keeping a physical distance. As a result, people's motivation to take precautions and willingness to help others can be increased.

### Limitations and Future Research

Our research has some limitations. Although we used realistic social distancing messages and tested our hypotheses with geographically and demographically varied participant groups in multiple languages and at different phases of the pandemic, our findings rely on self-reports obtained by online surveys. Therefore, we are limited in exploring participants' intentions rather than actual behaviors. To increase the external validity of our findings, testing the proposed hypotheses in a field study by evaluating citizens' actual responses to different messages would be fruitful. Although statistically sufficient, we also had a limited number of participants because of conducting online experiments; 119 participants in study 1, which used student respondents; and 202 and 201 participants in studies 1 and 3, which used an online participant pool. Additionally, in testing our hypotheses, we specifically measured “how persuasive and motivating” the messages were on willingness to self-isolate, keep a physical distance, and help others. Using more direct measures of the dependent variables could reduce the intention–behavior gap that might have occurred.

Across three studies, we manipulated prosocial vs. self-interested motives by using different pronouns (“our” vs. “your”) in the message. In study 2, we added people icons as visuals to the flier design to strengthen the message type manipulation. Specifically, one (four) person icon(s) was (were) used in self-interested (prosocial) message condition. Importantly, people icons were not used in other studies, and the results were replicated. Also, we asked participants to write their thoughts about the message in studies 1 and 2. Although this procedure is widely employed in experimental research (Rucker et al., 2011), people do not get asked to elaborate on their thoughts about a public health message in real life. However, they often get exposed to a message multiple times and hence are likely to process the message better compared to an experimental setting where only one exposure is provided. Regardless, we believe that study 3, which presented messages with different pronouns without using any manipulation strengthening methods, provides a more stringent and realistic test of the proposed effects. Moreover, while we used words and visuals in the form of written flyers to manipulate message type, future research can explore whether our findings will apply to spoken language (rather than written language), by manipulating the framing of health professionals' speeches (in which case the participants will hear rather than read the messages).

Further research can explore whether using descriptive norm appeals in a message (i.e., mentioning how most people behave in a situation; Cialdini et al., 1991) can influence persuasiveness of a social distancing message. Research in diverse domains has shown that knowing about others' actions in similar situations can significantly impact how an individual conforms to the descriptive norm (e.g., Goldstein et al., 2008). Accordingly, it is worth exploring how highlighting that the majority of citizens obey the physical distancing measures in the message can increase individuals' tendency to comply.

Finally, while we specifically focus on social distancing measures in this research, our findings may generalize to the communication of other preventive health measures, such as maintaining personal hygiene, washing hands frequently, or wearing masks. Further studies may test whether our hypotheses will similarly influence the interpretation of different communication messages that aim to limit the spread of epidemic diseases.

## Data Availability Statement

The raw data supporting the conclusions of this article will be made available by the authors, without undue reservation.

## Ethics Statement

The studies involving human participants were reviewed and approved by Ozyegin University Human Research Ethics Board. The patients/participants provided their written informed consent to participate in this study.

## Author's Note

Covid-19 outbreak has become a global health crisis for the past year. It has severely affected millions of people by impairing their physical and mental health. Keeping social distance and helping others in need are two important means of fighting the pandemic. Motivated by this, we test the evaluations of social distancing messages that use different appeals (self-interested vs. prosocial) across three studies. Our findings show that implying everyone's well-being, rather than the individual's own well-being, in the message increases persuasiveness of the message to stay at home and keep a physical distance from others, and also enhances motivations to help others in need. These effects are higher for those with a lower fear of coronavirus and lower locus of control as the message motivates these individuals to feel a joint responsibility to protect from the pandemic. Our results provide solid and quickly implementable guidelines for policymakers by responding to urgent calls on filling the covid-19 knowledge gaps in communicating health messages to the public in the most effective ways.

## Author Contributions

All authors listed have made a substantial, direct and intellectual contribution to the work, and approved it for publication.

## Conflict of Interest

The authors declare that the research was conducted in the absence of any commercial or financial relationships that could be construed as a potential conflict of interest.
