# Supplementary figures and images for: Message Framing Effects on Individuals' Social Distancing and Helping Behavior During the COVID-19 Pandemic
Source: Front Psychol. 2021 Mar 22;12:579164. doi: 10.3389/fpsyg.2021.579164 (PMC8019916; doi:10.3389/fpsyg.2021.579164)

## APPENDIX

### Study 1 Stimuli

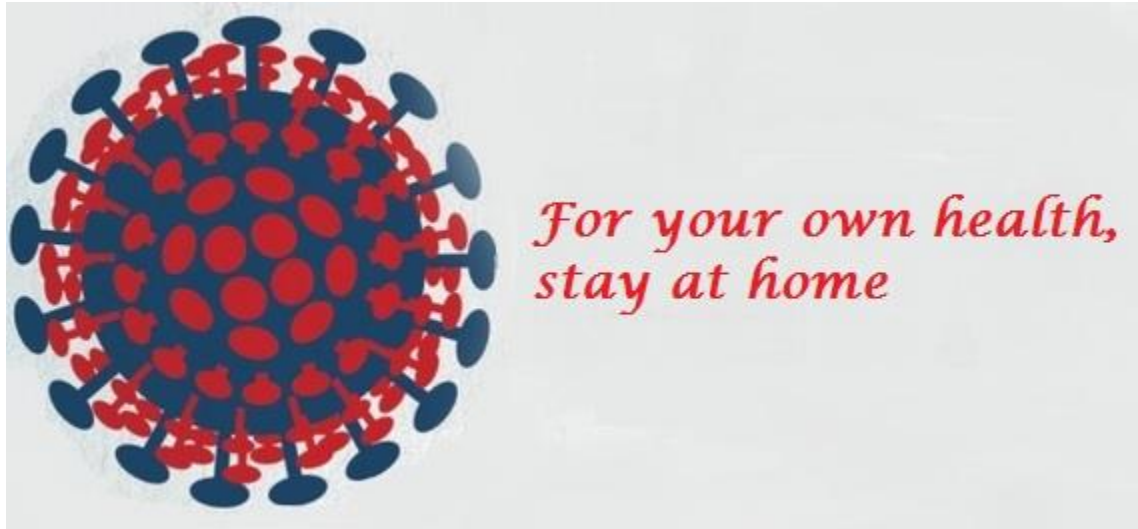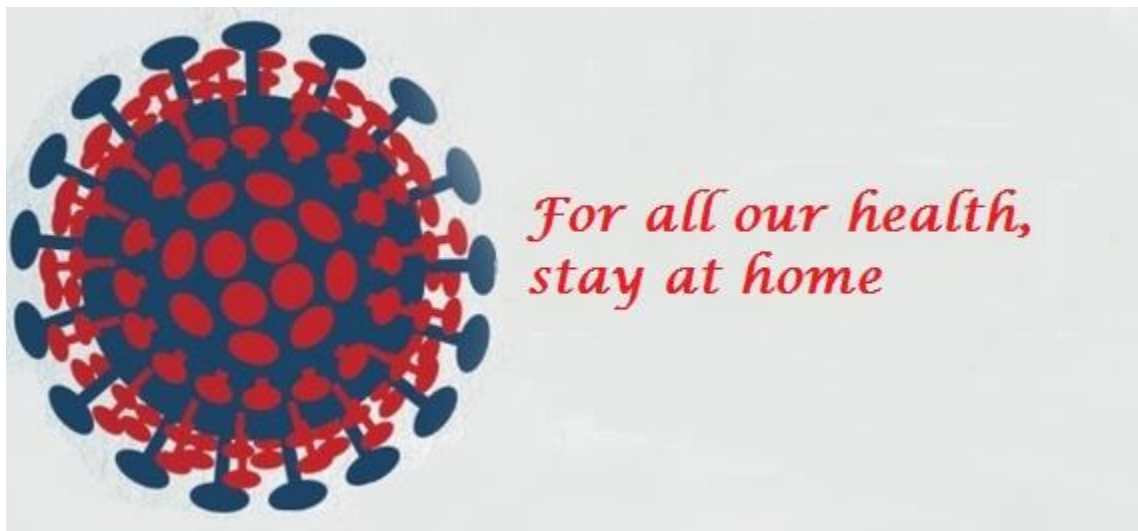

## Study 2 Stimuli

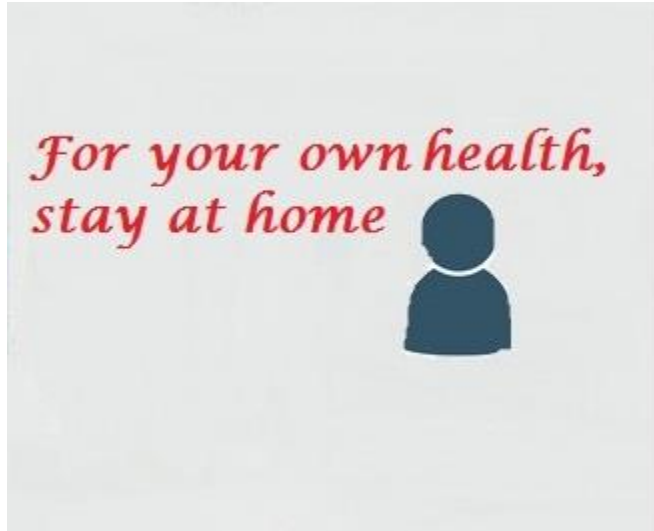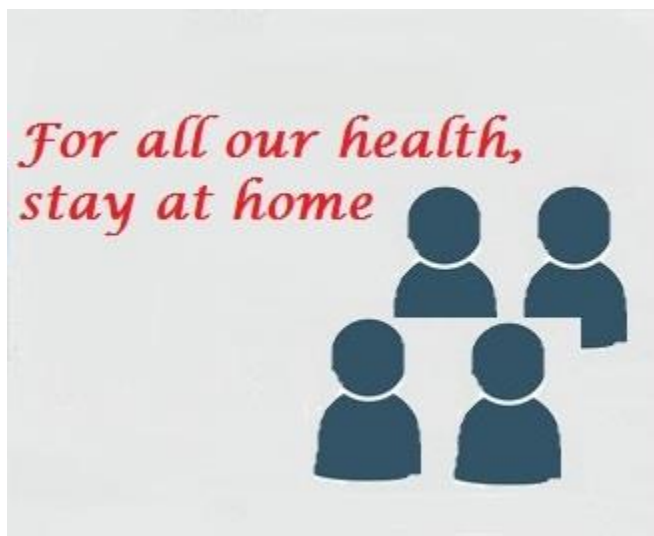

### Study 3 Stimuli

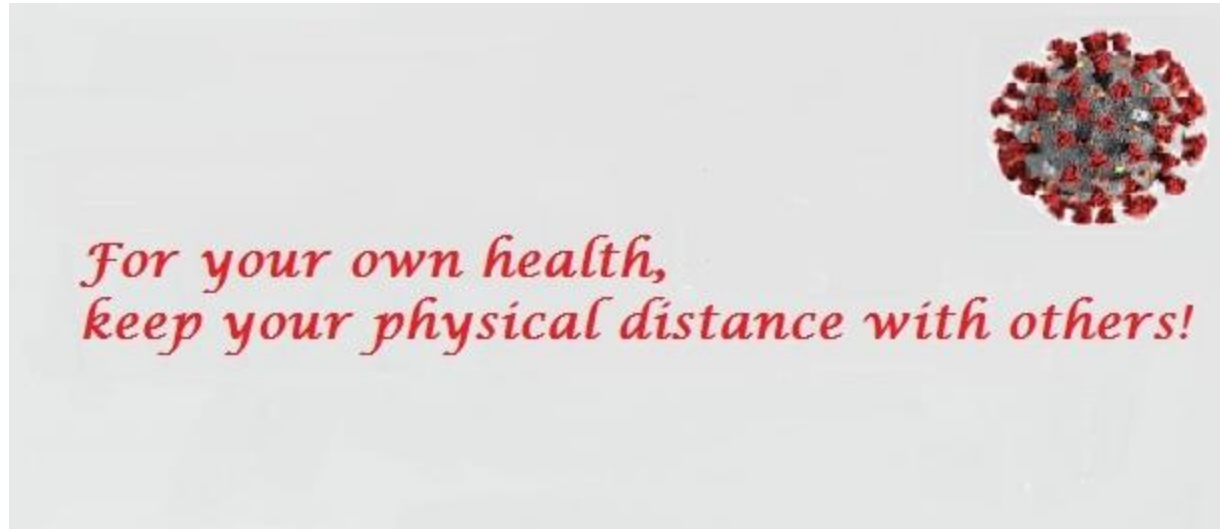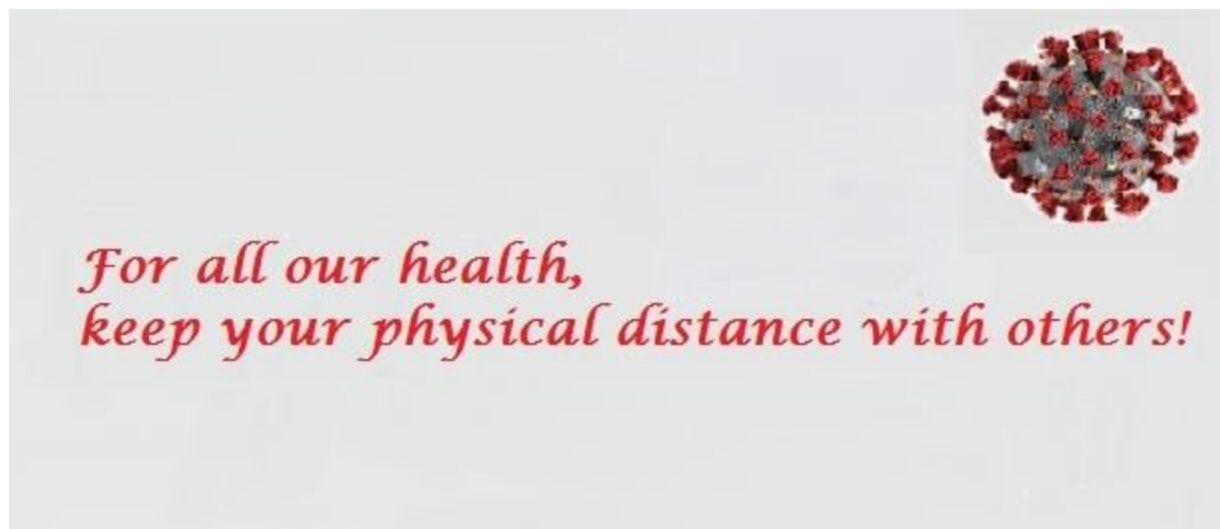

Supplement: Supplementary file 1 [file Data_Sheet_1.pdf]
